# Intensity-dependent modulation of cortical somatosensory processing during external, low-frequency peripheral nerve stimulation in humans

**DOI:** 10.1152/jn.00511.2021

**Published:** 2022-05-25

**Authors:** Danielle Hewitt, Alice Newton-Fenner, Jessica Henderson, Nicholas B. Fallon, Christopher Brown, Andrej Stancak

**Affiliations:** ^1^Department of Psychological Sciences, University of Liverpool, Liverpool, United Kingdom; ^2^Institute for Risk and Uncertainty, University of Liverpool, Liverpool, United Kingdom

**Keywords:** EEG, long-term depression, low-frequency stimulation, pain, somatosensation

## Abstract

External low-frequency peripheral nerve stimulation (LFS) has been proposed as a novel method for neuropathic pain relief. Previous studies have reported that LFS elicits long-term depression-like effects on human pain perception when delivered at noxious intensities, whereas lower intensities are ineffective. To shed light on cortical regions mediating the effects of LFS, we investigated changes in somatosensory-evoked potentials (SEPs) during four LFS intensities. LFS was applied to the radial nerve (600 pulses, 1 Hz) of 24 healthy participants at perception (1 times), low (5 times), medium (10 times), and high intensities (15 times detection threshold). SEPs were recorded during LFS, and averaged SEPs in 10 consecutive 1-min epochs of LFS were analyzed using source dipole modeling. Changes in resting electroencephalography (EEG) were investigated after each LFS block. Source activity in the midcingulate cortex (MCC) decreased linearly during LFS, with greater attenuation at stronger LFS intensities, and in the ipsilateral operculo-insular cortex during the two lowest LFS stimulus intensities. Increased LFS intensities resulted in greater augmentation of contralateral primary sensorimotor cortex (SI/MI) activity. Stronger LFS intensities were followed by increased α (alpha, 9–11 Hz) band power in SI/MI and decreased θ (theta, 3–5 Hz) band power in MCC. Intensity-dependent attenuation of MCC activity with LFS is consistent with a state of long-term depression. Sustained increases in contralateral SI/MI activity suggests that effects of LFS on somatosensory processing may also be dependent on satiation of SI/MI. Further research could clarify if the activation of SI/MI during LFS competes with nociceptive processing in neuropathic pain.

**NEW & NOTEWORTHY** Somatosensory-evoked potentials during low-frequency stimulation of peripheral nerves were examined at graded stimulus intensities. Low-frequency stimulation was associated with decreased responsiveness in the midcingulate cortex and increased responsiveness in primary sensorimotor cortex. Greater intensities were associated with increased midcingulate cortex θ band power and decreased sensorimotor cortex α band power. Results further previous evidence of an inhibition of somatosensory processing during and after low-frequency stimulation and point toward a potential augmentation of activity in somatosensory processing regions.

## Graphical abstract

**Figure:**
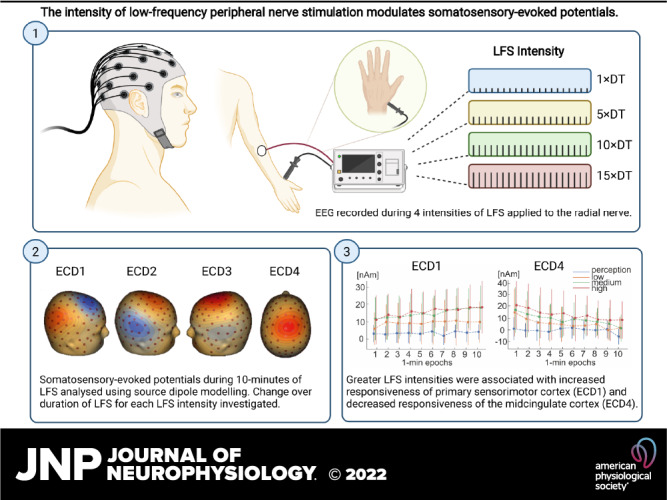

## INTRODUCTION

Neuropathic pain, defined as pain caused by a lesion or disease in the somatosensory system (1), is characterized by spontaneous pain, heightened pain sensitivity, and sensory loss (2, 3). Neuropathic pain syndromes affect ∼7%–10% of the general population (4), with considerable impacts on quality of life and functioning (5). First-line pharmacological treatments for neuropathic pain are associated with modest efficacy and adverse side effects (6–8), therefore, the development of new, effective treatments is of vital importance.

External, low-frequency peripheral nerve stimulation (LFS) has been proposed as a novel neurostimulation method for intractable neuropathic pain syndromes (9, 10). Long-term depression (LTD) of synaptic efficacy is theorized as the neurophysiological mechanism underlying LFS (11). LTD has been demonstrated at many sites in the central nervous system and can be induced in the nociceptive system after repetitive LFS (∼1–2 Hz) of Aδ fibers in the spinal dorsal horn both in vitro (11–14) and in vivo (15). Conversely, high-frequency stimulation (HFS, ∼100 Hz) of primary afferent fibers induces long-term potentiation of Aδ (16) and C-fiber responses (17–20), and may be a mechanism for pain amplification in acute and chronic pain states (21, 22). Long-term potentiation in the dorsal horn can be inhibited and reversed with LFS (11, 14, 15); therefore, LFS has important implications for our understanding of neuropathic pain.

Alterations in synaptic plasticity cannot be directly measured in humans, although changes in pain ratings and pain-related neural activity after LFS have been interpreted as indirect correlates of nociceptive LTD. In these studies, preferential activation of Aδ fibers through the skin is assumed with small diameter electrodes that deliver high current densities (23–27). LFS of peripheral nerve fibers in humans is associated with a sustained, homotopic decrease in perceived pain to noxious electrical stimuli (28–31) and a reversal of experimentally induced hyperalgesia (28, 32). LFS has been shown to decrease the amplitude of somatosensory-evoked potentials (SEPs) recorded with electroencephalography (EEG) during noxious electrical test stimuli (25, 29, 33). Thus, LFS appears to elicit strong effects on somatosensory processing in humans.

Although the poststimulation effects of LFS on nociceptive processing are well established, few studies have investigated neural activation changes during the time course of LFS. We recently demonstrated in healthy volunteers that LFS is associated with source activity in the primary sensorimotor cortex (SI/MI), bilateral operculo-insular cortex, and midcingulate cortex (MCC) (34). By recording SEPs over the duration of LFS, we showed a linear decrease in SEP amplitude in the MCC and ipsilateral operculo-insular cortex. These findings are in line with previous studies demonstrating that LFS is associated with a linear decrease in excitatory postsynaptic potentials in vitro (11) and gradual decreases in pain ratings during LFS conditioning in humans (25, 28, 35, 36). We also showed that LFS was associated with poststimulation increases in 8–12 Hz α and 16–24 Hz β band power in electrodes overlying contralateral operculo-insular and sensorimotor cortices (34), consistent with cortical inhibition and idling in regions that mediate sensory perception (37, 38). Taken together, this indicates that LFS has lasting, inhibitory effects on sensory processing that may be mediated by the MCC and operculo-insular cortex.

Induction of LTD has been shown to be dependent on LFS intensity. It is well established that LFS of afferent fibers at noxious intensities sufficient to activate Aδ fibers induces sustained LTD, whereas lower intensities activating Aβ fibers produce no inhibition (39) or only a transient decrease in synaptic transmission (11, 15). Likewise, in human studies, maximal reduction in SEP amplitude and acute pain perception have been observed after 1,200 pulses of 1 Hz stimulation at four times pain threshold, corresponding to 15 times detection threshold (29). Conversely, lower intensities at one time and two times pain threshold produced a smaller reduction in SEPs and pain ratings. In our recent study, we used a distinctly uncomfortable but not painful LFS intensity that may have been insufficient to activate Aδ fibers (34). Thus, to examine if reduced amplitude of SEPs estimated to be generated by the MCC and operculo-insular cortex during LFS are specific to LTD, it is crucial to compare the effects of noxious intensities of LFS to stimulation at nonpainful intensities.

To shed light on the cortical regions mediating the suppression of activity during nonpainful and noxious intensities of LFS, we investigated changes in SEPs and poststimulation resting oscillations with four intensities of continuous LFS in healthy human volunteers. Based on previous evidence that 15 times detection threshold is sufficient to elicit attenuation of SEPs (29), the four intensities used were 1 times, 5 times, 10 times, and 15 times detection threshold. Using source analysis, we characterized the locations of sources contributing to the SEPs and how activity in the sources changed over the duration of LFS. We predicted that LFS would be associated with a linear reduction of SEP amplitude in sources originating in the MCC and ipsilateral operculo-insular cortex, and that the greatest reduction in SEP amplitude would be found in these sources at the highest LFS intensity. We secondly investigated whether the cortical regions showing activation changes over the period of LFS would similarly show poststimulation modulation of resting oscillatory activity. It was predicted that greater intensities of LFS would be associated with greater increases in α and β band power and decreases in θ band power in source signals generated in operculo-insular and cingulate cortex.

## MATERIALS AND METHODS

### Subjects

Twenty-eight healthy subjects (14 females) with no history of chronic pain or neurological conditions were recruited from a pool of undergraduate and postgraduate students at the University of Liverpool. Four subjects were excluded during data collection: three as they could not tolerate the electrical stimulation and one due to excessive movement artifacts. The final sample included 24 participants (11 females, 22 right handed) with a mean age of 25 ± 4.2 yrs (mean ± SD). The procedure was approved by the Research Ethics Committee of the University of Liverpool, and all participants gave written informed consent at the start of the experiment in accordance with the Declaration of Helsinki. Participants were reimbursed with £20 for their time on completion of the study.

### Experimental Protocol

Experimental procedures were carried out in a single 2-h session in the Eleanor Rathbone Building, University of Liverpool. Electrical detection thresholds were determined before the start of the experiment using the method of limits. During the experiment, participants were seated in a dimly lit room with a 19-in. LCD monitor in front of them. Participants were instructed to keep their eyes open and look straight ahead for 4 min during the recording of resting EEG.

LFS was applied to the dorsal aspect of the hand during four conditions, each modulated by LFS intensity: perception (detection threshold), low (5 times detection threshold), medium (10 times detection threshold), and high (15 times detection threshold). Each block of LFS lasted ∼10 min. Participants were randomly assigned one of six possible block orders with each block order represented four times. Presentation of LFS stimuli was controlled with Cogent 2000 (University College London, London, UK) in MATLAB 2010 (The MathWorks, Inc.). After each block of LFS, resting EEG was recorded for 4 min while participants looked straight ahead with eyes open, followed by a break of 6 min to ensure that participants remained alert and to allow the researcher to check electrode impedances.

Participants’ ratings of electrical stimulation were collected at the start of the experiment and after each block of stimulation by applying a single electrical stimulus to the test site using the conditioning LFS electrode. Participants were informed that the intensity of test stimulation was the maximum level that they would receive during the experiment, but that their perception of the stimulus may change throughout the study. Participants were asked to rate the painfulness of the test stimuli on a verbal numeric rating scale from 0 (no pain) to 10 (maximum pain), where 3 indicated pain threshold, and unpleasantness from 0 (not at all unpleasant) to 10 (maximum unpleasantness).

#### Electrical stimulation.

LFS was applied to the skin in the region of the radial nerve of the left hand using a pen electrode with 4 mm diameter cathode (Compex Motor Point Pen, UK) and a distal 5-cm^2^ flat electrode by the olecranon process that served as an anode, controlled by a Digitimer DS7A constant current stimulator (Digitimer, UK) and MATLAB 2010. Electrodes using a small cathode area such as pin, concentric, or pen electrodes been designed to preferentially activate Aδ fibers without coactivation of Aβ fibers (23, 24, 40–42). To the best of our knowledge, the proportion of Aδ and Aβ fiber involvement during low- or high-intensity LFS is not known.

Stimulus detection threshold was determined at the start of the experiment using the method of limits, where single electrical stimuli were delivered to the test site in descending and ascending steps of 0.02 milliamperes (mA) to establish the lowest threshold at which participants could perceive the stimuli (detection threshold). During the experiment, LFS was applied at four intensities calculated as multiples of detection threshold: 1 times, 5 times, 10 times, or 15 times detection threshold, with the highest intensity in line with previous studies using LFS (29, 43). If participants found the high-intensity condition to be intolerable, intensity was reduced to a painful but tolerable level, and medium- and low-intensity conditions were modified to 66% and 33% of the high intensity, respectively. Mean stimulus intensities were as follows: perception 0.12 ± 0.04 mA, low 0.63 ± 0.32 mA, medium 1.19 ± 0.39 mA, and high 1.78 ± 0.59 mA. An independent-samples *t* test showed that electrical detection threshold was higher in male (M = 0.14 mV, SD = 0.04) compared with female participants (M = 0.11 mV, SD = 0.04) [*t*(22) = 2.09, *P* = 0.048]. Each block of LFS consisted of 600 pulses delivered at a frequency of 1 Hz, pulse width of 1 ms, and duration of 1 ms. The total number of pulses was reduced in contrast to previous studies with LFS to reduce the burden on participants, based on evidence that these parameters are sufficient to elicit a prolonged suppression of SEPs and pain ratings to electrical test stimuli (29). In addition, single electrical test stimuli were applied to the test site at the high intensity (15 times detection threshold) at baseline and after each LFS block using the same electrode as the conditioning stimulus.

#### EEG acquisition.

Continuous EEG was recorded using a 129-channel Geodesics EGI System (Electrical Geodesics, Inc., Eugene, OR) with a sponge-based HydroCel Sensor Net. The sensor net was aligned with respect to three anatomical landmarks of two preauricular points and the nasion. Electrode-to-skin impedances were kept below 50 kΩ throughout the experiment. A recording bandpass filter was set at 0.001–200 Hz with a sampling rate of 1,000 Hz. Electrode Cz was used as a reference electrode during the recordings.

### Analysis of LFS Ratings

Mean ratings of pain and unpleasantness to electrical test stimuli in each condition were calculated for each participant. To assess differences in pain and unpleasantness ratings of test stimuli after each LFS intensity, 1 × 4 repeated-measures analyses of covariance (ANCOVA) were computed using SPSS v. 27 (IBM Inc.) separately for pain and unpleasantness, with an independent variable of “LFS Intensity” (perception, low, medium, and high intensity) and baseline scores as a covariate. Post hoc *t* tests were used where appropriate to investigate significant main effects.

### EEG Data Analysis

EEG data were processed using BESA v. 6.1 (MEGIS GmbH, Germany). Data were filtered using 1 Hz high-pass and 70 Hz low-pass filters, with a notch filter of 50 Hz ± 2 Hz. EEG data were spatially transformed to reference-free data using common average method (44). Oculographic and electrocardiographic artifacts were removed with principal component analysis (45). Data were visually inspected for movement and muscle artifacts. Trials containing artifacts were marked and excluded from further analysis. Electrode channels with large artifacts were interpolated to a maximum of 10% of electrodes.

#### Analysis of SEPs during LFS.

SEPs were evaluated during 10 min of LFS applied to the radial nerve. All 600 SEP responses over the 10-min recording were divided into 10 1-min intervals of 60 responses each. SEP responses in each 1-min interval were averaged in the epoch −100 ms prestimulus to 900 ms poststimulus. The baseline period ranged from −100 to −5 ms before stimulus onset, and the stimulus artifact window was defined as −4 to 10 ms poststimulus. Data were filtered during averaging from 1 to 45 Hz. The mean number of epochs containing SEP responses accepted for further analysis was 56.99 ± 0.27 (means ± SE). A repeated-measures ANOVA showed that the average number of accepted epochs was significantly different between LFS intensities [*F*(3,69) = 2.86, *P* = 0.043] due to fewer accepted epochs during high intensity (56.45 ± 0.41; *P* = 0.015) compared with perception intensity LFS (57.26 ± 0.26). Accepted epochs were significantly different over the 10-min duration of LFS [*F*(9,207) = 5.69, *P* < 0.001]. Pairwise comparisons showed a significant increase in the number of accepted epochs in *minutes 2*–*10* compared with *minute 1* (*P* < 0.05). The interaction between LFS intensity and duration was not significant (*P* > 0.05).

#### Source dipole modeling.

Source dipole modeling of SEPs was performed using BESA v. 6.1 (MEGIS GmbH, Germany). The source dipole model was constructed using a sequential strategy as used in our previous study (34), in which equivalent current dipoles (ECDs) were fitted consecutively from the first peak in global field power (46–49). Due to the presence of a large stimulus artifact from LFS, dipoles were fitted between 30 ms and 900 ms poststimulus. When residual variance was not reduced by adding another dipole, the fitting procedure was terminated. Classical LORETA analysis recursively applied (CLARA) (50) was used as an independent source localization method to confirm the location of ECDs.

To evaluate the effect of LFS duration on SEPs, the grand average source dipole model was used to compute individual subject source waveforms for all 10 1-min intervals of LFS. A permutation analysis with 2,000 repetitions, implemented in the *statcond.m* program in EEGLab (51), was utilized to identify time intervals showing significant main effects and interactions of LFS duration and intensity (52). This method provides a data-driven approach to test the effects across all timepoints while controlling for multiple comparisons with no loss in statistical power. Time intervals surrounding source waveform peaks were defined for each ECD (ECD1 35–75 ms, ECD2 100–150 ms, ECD3 105–155 ms, and ECD4 150–260 ms). Intervals surrounding ECD peaks that exceeded a predefined threshold on the calculated *P* values (corrected *P* < 0.001) for the main effect or interactions of LFS duration and intensity were selected for further analysis. Source dipole moments in intervals deemed significant by permutation tests were entered into individual 4 × 10 repeated-measures ANOVAs involving the four LFS intensities (perception, low, medium, and high) and each 10-min interval (*minutes 1*–*10*) for each ECD. The Huynh–Feldt correction was used where necessary to tackle a violation of the sphericity assumption, denoted by ε.

#### Linear regression analysis.

Linear regression analysis was conducted to analyze if source dipole moments showed a systematic decrease in amplitude over time, consistent with LTD. Linear regression analysis was carried out in every subject and each level of LFS intensity, with LFS duration as a predictor variable and source dipole moments as dependent measures. As the regression analysis assessed the slope of change in ECD amplitude over time, only source dipole moments that showed a significant main effect of time were included.

The resultant regression coefficients from linear regression analysis for all subjects were entered into one sample *t* tests to examine if any of the four LFS intensities showed a significant difference from zero. Regression coefficients showing a significant difference from zero were compared individually for each ECD using repeated-measures ANOVAs to investigate changes across the four LFS intensities.

#### Resting EEG analysis.

To evaluate the effect of LFS intensity on ongoing oscillatory activity, the grand average source dipole model was used to compute individual subject source waveforms in resting EEG recorded before LFS and after each LFS intensity. Each ECD in the source dipole model yielded, after back projection onto resting-state EEG data using the surrogate model method, a continuous source signal sampled at 256 Hz. Individual continuous source signals for all LFS intensities were exported to MATLAB 2019b for spectral analysis. Power spectral density was estimated using Welch’s method in the frequency range 0–128 Hz in nonoverlapping 1-s segments. Data were smoothed using a Hamming window. For each subject, time samples containing artifacts were removed from the data before spectral analysis, and data were trimmed to the length of the shortest condition to avoid differences in data length affecting the results. The mean duration of resting EEG data for which power spectra were estimated was 231 ± 7 s (means ± SD).

Only frequency components between 1 and 40 Hz were considered for statistical analysis. Mean absolute power values were transformed using a decadic logarithmic transform. One-way ANOVAs were computed to investigate the effect of LFS intensity (perception, low, medium, and high) on spectral power in all frequencies from 1 to 40 Hz in each of the four ECDs. Analyses were carried out on all frequencies from 1 to 40 Hz to investigate the specific frequency components showing changes in resting oscillatory activity. Resting EEG recorded before LFS was not included in statistical analyses as the order of this condition was not permuted. To control for Type I error likely to occur due to the large number of ANOVAs, the resulting statistical probability values were subject to permutation analysis using the *statcond.m* program with 2,000 permutations. Frequency components that exceeded a predefined threshold (*P* < 0.05) for the main effect of LFS intensity were selected for further analysis. Pairwise comparisons were computed to investigate the direction of effects.

Next, Pearson’s correlations were conducted in MATLAB to analyze the relationship between linear regression slopes (β) for change in source dipole amplitude during LFS at each of the four LFS intensities, and change in resting oscillatory band-power after stimulation. Correlations were computed individually for source waveforms showing significant differences from zero, and resting band power after each intensity of LFS in the corresponding source waveforms. To correct for individual variations in resting band power, a baseline correction was implemented by subtracting spectral power before stimulation from absolute power after each of the four LFS intensities.

## RESULTS

### Mean Ratings of Electrical Test Stimuli

Mean pain ratings of test stimuli before LFS were 4.5 ± 1.3 on a scale of 0–10, where 3 indicated pain threshold. Mean unpleasantness scores for test stimuli before LFS were 6.04 ± 1.9 on a scale of 0–10. Mean pain and unpleasantness ratings for test stimuli did not significantly vary with LFS intensity [*F*(3,66) = 0.4, *P* > 0.05; *F*(3,66) = 0.23, *P* > 0.05].

### Source Dipole Model

**Figure 1.**
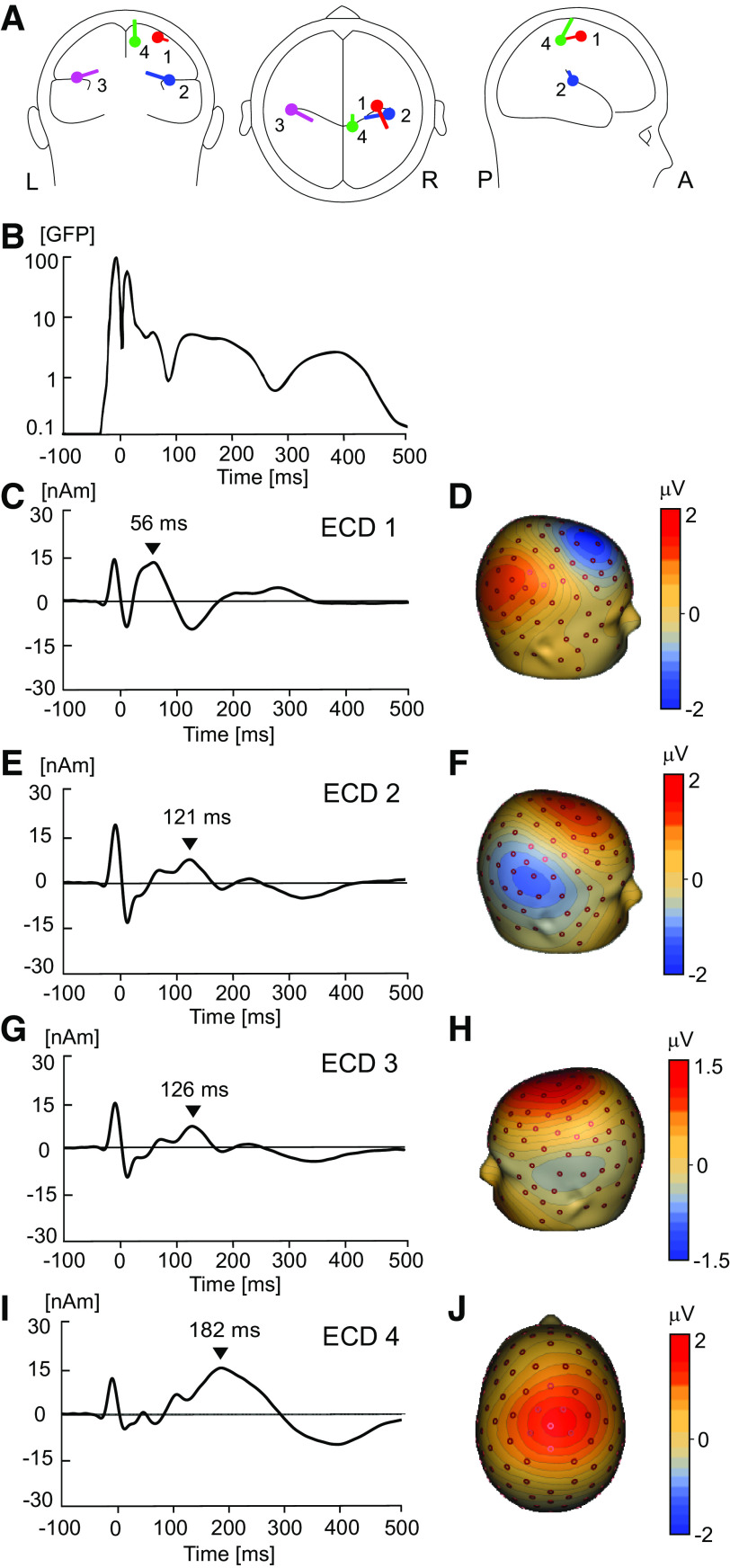
Source dipole model of somatosensory-evoked potentials (SEPs) during 10 min of low-frequency peripheral nerve stimulation (LFS). *A*: location and orientation of four equivalent current dipoles (ECDs) in transparent glass brains determined using a data-driven sequential strategy. *B*: global field power (GFP) of grand average electroencephalography (EEG) potentials averaged over all participants (*n* = 24, 11 females) and all 1-min epochs over the 10-min duration of LFS. *C*: time course of ECD1 with a positive peak latency of 56 ms. *D*: potential maps of the P56 component showing a positive potential maximum in parietal area and a negative maximum in contralateral central and frontal electrodes, consistent with a tangential dipole located in primary sensorimotor cortex (SI/MI). *E*: time course of ECD2 with a positive peak latency of 121 ms. *F*: potential maps of the N2 component showing a negative maximum in contralateral temporal electrodes, suggesting the presence of a radial dipole located in the contralateral operculo-insular cortex. *G*: time course of ECD3 with a positive peak latency of 126 ms. *H*: potential maps of the N2 component showing a negative maximum in ipsilateral temporal electrodes, suggestive of a radial dipole located in the operculo-insular cortex. *I*: ECD4 time course with a positive peak latency of 182 ms. *J*: potential maps of the P182 component showing a positive potential on the vertex, consistent with a radially orientated current dipole located in the midcingulate cortex (MCC).

Changes in SEPs over the 10 min of LFS were analyzed using source dipole analysis. The four-dipole model accounted for 83.62% of the variance in the SEP (Fig. 1, *A*and *B*). Addition of a fifth dipole with free orientation and location to the source dipole model resulted in an anterior dipole close to ECD4, which did not improve the residual variance; therefore, the four-dipole solution was used. CLARA method was used to verify the location of fitted ECDs. Results were highly convergent between the two models, with a maximum discrepancy of ∼2 mm between ECD3 and the maxima of the CLARA cluster.

ECD1 was located in the contralateral SI/MI (approximate Talairach coordinates: *x* = 30 mm, *y* = −20 mm, *z* = 60 mm). This source waveform had a negative peak at 56 ms (Fig. 1, *C* and *D*). ECD2 was located in the upper bank of the right Sylvian fissure comprising the secondary somatosensory cortex (approximate Talairach coordinates: *x* = 40 mm, *y* = −23 mm, *z* = 17 mm). The source waveform had a positive peak in frontal electrodes at 121 ms (Fig. 1, *E* and *F*) . ECD3 was located in the left Sylvian fissure symmetrical to ECD2, consistent with the ipsilateral secondary somatosensory cortex (approximate Talairach coordinates: *x* = −45 mm, *y* = −19 mm, *z* = 20 mm). The source waveform of ECD3 (Fig. 1, *G* and *H*) waveform had a positive peak in frontal electrodes at 126 ms. Finally, ECD4 was located in the medial parietal cortex involving the mid- and posterior cingulate cortex (approximate Talairach coordinates: *x* = 8 mm, *y* = −42 mm, *z* = 54 mm). The ECD4 source waveform had a positive peak across the vertex with a latency of 182 ms (Fig. 1, *I* and *J*).

#### Changes in SEPs during LFS.

**Figure 2.**
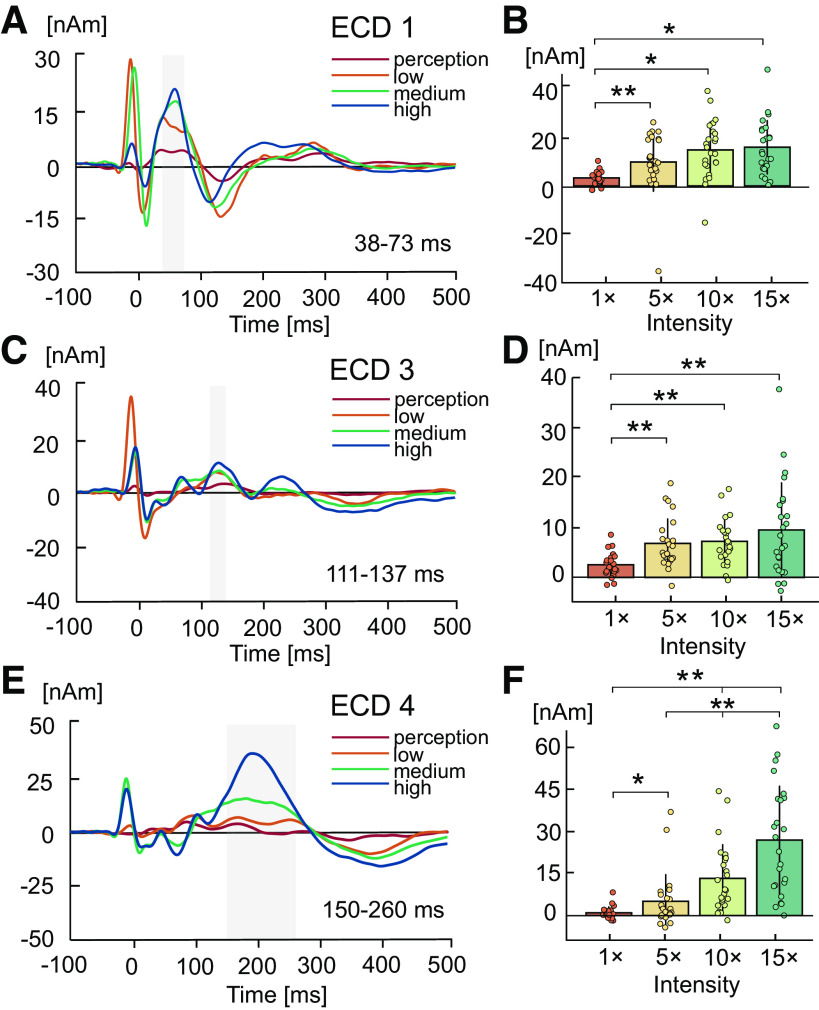
Grand average changes in somatosensory-evoked potentials (SEPs) at varying intensities of low-frequency stimulation (LFS). One-way ANOVAs showed a statistically significant amplitude modulation by LFS intensity for equivalent current dipole (ECD)1 (*A*), ECD3 (*C*), and ECD4 (*E*). Shaded bars indicate significant time intervals around source waveform peaks. Bar graphs show the mean amplitudes and standard deviation of ECD1 (*B*), ECD3 (*D*), and ECD4 (*F*) source waveforms during perception (1 times), low (5 times), medium (10 times), and high LFS intensities (15 times detection threshold) in corresponding time intervals. Individual subject data points averaged over single trials are overlaid for each condition. Pairwise comparisons were computed to identify contrasts that exceeded significance at *P < 0.05 or **P < 0.001. *n* = 24 (11 females).

Figure 2, *A*, *C*, and *E* shows the time intervals around source waveform peaks showing a significant effect of LFS intensity. Amplitude of ECD1 was significantly modulated by LFS intensity [*F*(3,69) = 15.57, *P* < 0.001, ${\text{η}}_{\text{p}}^{2}$ = 0.40; Fig. 2*B*]. Pairwise comparisons showed an increase in amplitude at low (*P* = 0.010), medium (*P* < 0.001), and high (*P* < 0.001) LFS intensities compared with the lowest perception level. ECD2 showed no statistically significant changes during LFS (*P* > 0.05). ECD3 was significantly modulated by LFS intensity [*F*(3,69) = 10.68, *P* < 0.001, ε = 0.58, ${\text{η}}_{\text{p}}^{2}$ = 0.32; Fig. 2*D*], with an increase in amplitude at low, medium, and high LFS intensities compared with the lowest perception level (all *P* < 0.001). ECD4 also showed a significant increase in amplitude with LFS intensity [*F*(3,69) = 33.76, *P* < 0.001, ε = 0.56, ${\text{η}}_{\text{p}}^{2}$ = 0.60; Fig. 2*F*] due to a significant increase in amplitude at greater LFS intensities (perception vs. low, *P* = 0.028, all other comparisons, *P* < 0.001). In addition, the statistical test of trend components confirmed that amplitude increased linearly with greater LFS intensities for ECD1 [*F*(1,23) = 40.03, *P* < 0.001, ${\text{η}}_{\text{p}}^{2}$ = 0.64], ECD3 [*F*(1,23) = 14.04, *P* < 0.001, ${\text{η}}_{\text{p}}^{2}$ = 0.38] and ECD4 [*F*(1,23) = 46.67, *P* < 0.001, ${\text{η}}_{\text{p}}^{2}$ = 0.67] but not for ECD2 (*P* > 0.05).

**Figure 3.**
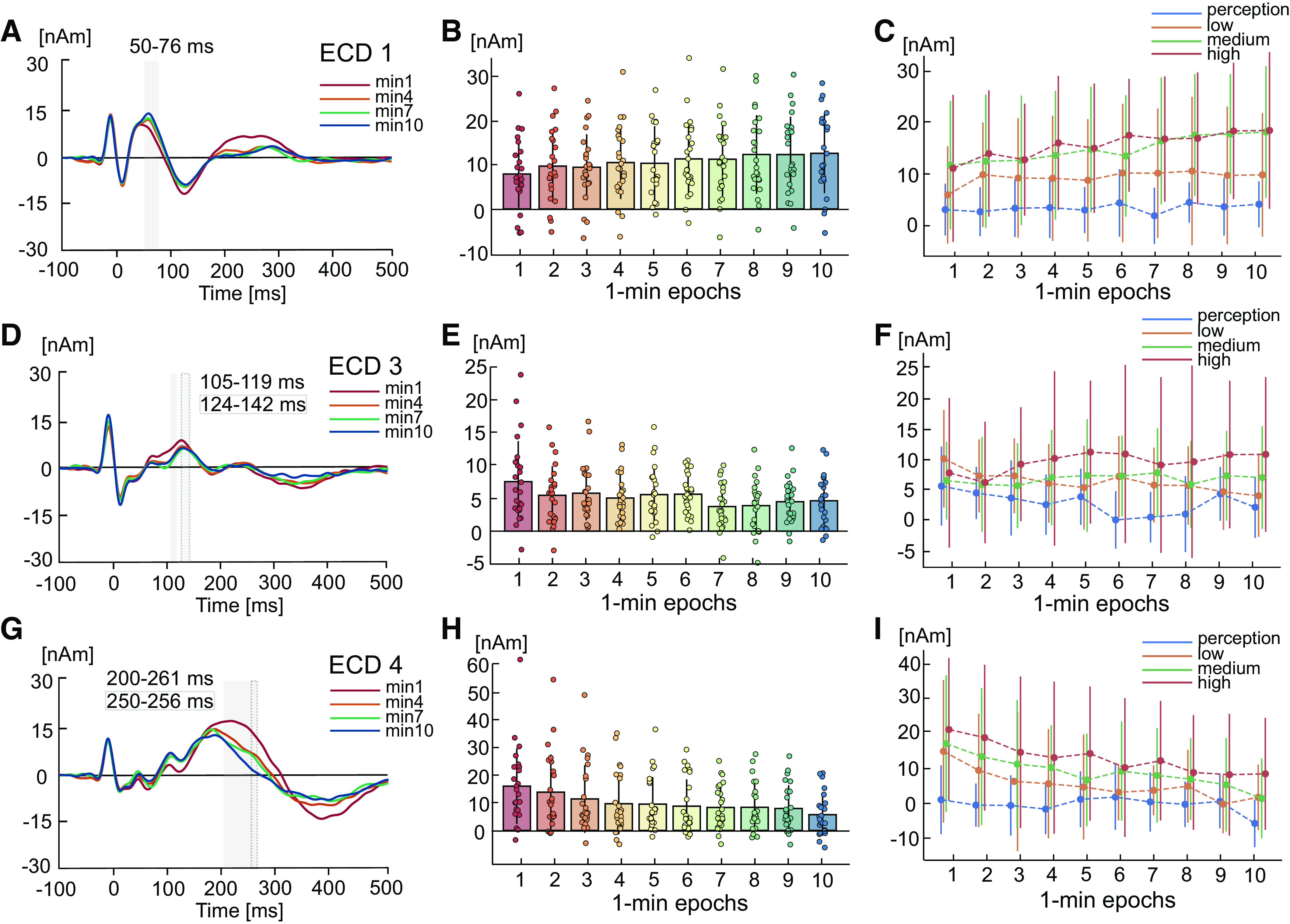
Grand average changes in somatosensory-evoked potentials (SEPs) during 10 min of low-frequency stimulation (LFS) and at varying LFS intensities. One-way ANOVAs showed a statistically significant amplitude modulation by LFS duration for equivalent current dipole (ECD)1 (*A*), ECD3 (*D*), and ECD4 (*G*). Source waveforms and standard deviations in *minute 1*, *minute 4*, *minute 7*, and *minute 10* are shown for illustrative purposes. Bars indicate time intervals around source waveform peaks showing a significant effect of LFS duration (shaded only) or an interaction between LFS duration and intensity (shaded with outline). Bar graphs show the mean amplitudes and standard deviation of ECD1 (*B*), ECD3 (*E*), and ECD4 (*H*) source waveforms in 1-min intervals over the 10-min duration of LFS, averaged over all LFS intensities. Individual subject data points averaged over single trials are overlaid for each condition. Simple contrasts were computed to identify contrasts that exceeded significance from LFS *minute 1*. Scatter graphs show mean amplitudes of ECD1 (*C*), ECD3 (*F*), and ECD4 (*I*) source waveforms during each of the four LFS intensities for all 1-min epochs of LFS. Error bars indicate standard deviation. *n* = 24 (11 females).

ECD1 amplitude was significantly modulated by LFS duration [*F*(9,207) = 9.08, *P* < 0.001, ε = 0.74, ${\text{η}}_{\text{p}}^{2}$ = 0.28; Fig. 3, *A* and *B*] in the interval 50–76 ms. Simple contrasts indicated that this effect was due to an increase in amplitude during *minute 2* (*P* = 0.006), *minute 3* (*P* = 0.031), *minute 5* (*P* = 0.007), and *minutes 4* and *6–10* (*P* < 0.001) compared with *minute 1*. The linear trend was also statistically significant [*F*(1,23) = 33.79, *P* < 0.001, ${\text{η}}_{\text{p}}^{2}$ = 0.60], indicating that ECD1 amplitude increased with successive LFS stimuli. ECD2 showed no statistically significant changes during LFS (*P* > 0.05). ECD3 was significantly modulated by LFS duration [*F*(9,207) = 4.95, *P* < 0.001, ε = 0.63, ${\text{η}}_{\text{p}}^{2}$ = 0.18; Fig. 3, *D* and *E*] in the latency epoch 105–119 ms, with a significant linear decrease in amplitude over time [*F*(1,23) = 10.12, *P* < 0.004, ${\text{η}}_{\text{p}}^{2}$ = 0.31]. Simple contrasts from *minute 1* indicated a significant decrease in amplitude during *minute 2* (*P* = 0.011), *minute 4* (*P* = 0.009), *minute 5* (*P* = 0.040), *minutes 7* and *8* (*P* = 0.003), *minute 9* (*P* = 0.007), and *minute 10* (*P* = 0.005). ECD4 also showed a significant linear decrease in amplitude with LFS duration [*F*(9,207) = 12.94, *P* < 0.001, ε = 0.67, ${\text{η}}_{\text{p}}^{2}$ = 0.36; *F*(1,23) = 20.40, *P* < 0.001, ${\text{η}}_{\text{p}}^{2}$ = 0.47; Fig. 3, *G* and *H*] in the interval 200–261 ms. Simple contrasts from *minute 1* showed a statistically significant decrease in amplitude during *minute 2* (*P* = 0.029), *minutes 3* and *4* (*P* < 0.001), *minute 5* (*P* = 0.002), and *minutes 6–10* (*P* < 0.001).

Figure 3, *A*, *D*, and *G* illustrates time intervals around source waveform peaks showing a significant interaction between LFS duration and intensity. ECD3 showed a statistically significant interaction between LFS duration and intensity in the window 124–142 ms [*F*(27,621) = 2.23, *P* = 0.003, ${\text{η}}_{\text{p}}^{2}$ = 0.09; Fig. 3*F*]. Repeated-measures ANOVAs showed that this effect was due to reductions in ECD3 amplitude over time both during perception [F(9,207) = 3.72, *P* < 0.001, ${\text{η}}_{\text{p}}^{2}$ = 0.14] and low LFS intensities [*F*(9,207) = 2.97, *P* = 0.002, ${\text{η}}_{\text{p}}^{2}$ = 0.11]; in contrast, there was no significant change in ECD3 amplitude during medium and high LFS intensities (*P* > 0.05). ECD4 showed a significant interaction between LFS duration and intensity in the window 250–256 ms [*F*(27,621) = 2.07, *P* = 0.012, ${\text{η}}_{\text{p}}^{2}$ = 0.08; Fig. 3*I*]. Repeated-measures ANOVAs showed that this was due to significant reductions in ECD4 amplitude over time during low [*F*(9,207) = 7.66, *P* < 0.001, ${\text{η}}_{\text{p}}^{2}$ = 0.25], medium [*F*(9,207) = 5.88, *P* < 0.001, ${\text{η}}_{\text{p}}^{2}$ = 0.20], and high [*F*(9,207) = 7.89, *P* < 0.001, ${\text{η}}_{\text{p}}^{2}$ = 0.26] LFS intensities, but not perception intensity (*P* > 0.05).

In summary, results point toward a modulation of ECD1 and ECD4 amplitude at greater LFS intensities, and decreased ECD3 amplitude during lower LFS intensities.

#### Linear regression slopes.

**Figure 4.**
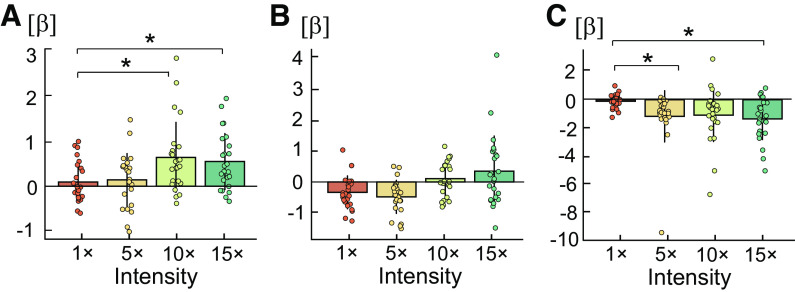
Mean linear regression coefficients (β) and standard deviation of the change in source dipole moment activity over the duration of low-frequency stimulation (LFS) for each LFS intensity: perception (1 times), low (5 times), medium (10 times), and high intensity (15 times detection threshold). Positive values indicate an ascending slope with increased amplitude over the duration of LFS, while negative values indicate a descending slope with decreased amplitude during LFS. Pearson’s correlations were conducted to analyze the relationship between regression slopes and equivalent current dipole (ECD) amplitude. *A*: ECD1 showed a significantly steeper positive slope at medium and high LFS intensities compared with perception intensity. *B*: regression coefficients in ECD3 did not significantly differ between LFS intensities. *C*: ECD4 showed a significantly steeper negative slope at low and high LFS intensities compared with perception intensity. *Contrasts that exceeded significance at *P* < 0.05. *n* = 24 (11 females).

Repeated-measures ANOVAs were computed to assess significant differences in linear regression slopes (β) between LFS intensities for source dipole moments showing a significant difference from zero in one-way ANOVAs: ECD1, ECD3, and ECD4. Mean regression coefficients for each intensity are shown in Fig. 4, *A*–*C*. ECD1 showed a statistically significant effect of LFS intensity [*F*(3,69) = 5.87, *P* = 0.001, ${\text{η}}_{\text{p}}^{2}$ = 0.20]. Pairwise comparisons indicated that this effect was due to a significantly steeper positive slope at medium (*P* = 0.044) and high LFS intensities (*P* = 0.012) compared with perception intensity (Fig. 4*A*). ECD3 showed a statistically significant effect of LFS intensity [*F*(3,69) = 3.10, *P* = 0.032, ${\text{η}}_{\text{p}}^{2}$ = 0.13], however pairwise comparisons between LFS intensities did not survive Bonferroni correction (*P* > 0.05) (Fig. 4*B*). ECD4 showed a statistically significant effect of LFS intensity [*F*(3,69) = 5.87, *P* = 0.001, ${\text{η}}_{\text{p}}^{2}$ = 0.20]. Pairwise comparisons indicated that this effect was due to a significantly steeper negative slope at low- (*P* = 0.044) and high LFS intensities (*P* = 0.007) compared with perception intensity (Fig. 4*C*).

### Oscillatory Changes in Source Signals after LFS

**Figure 5.**
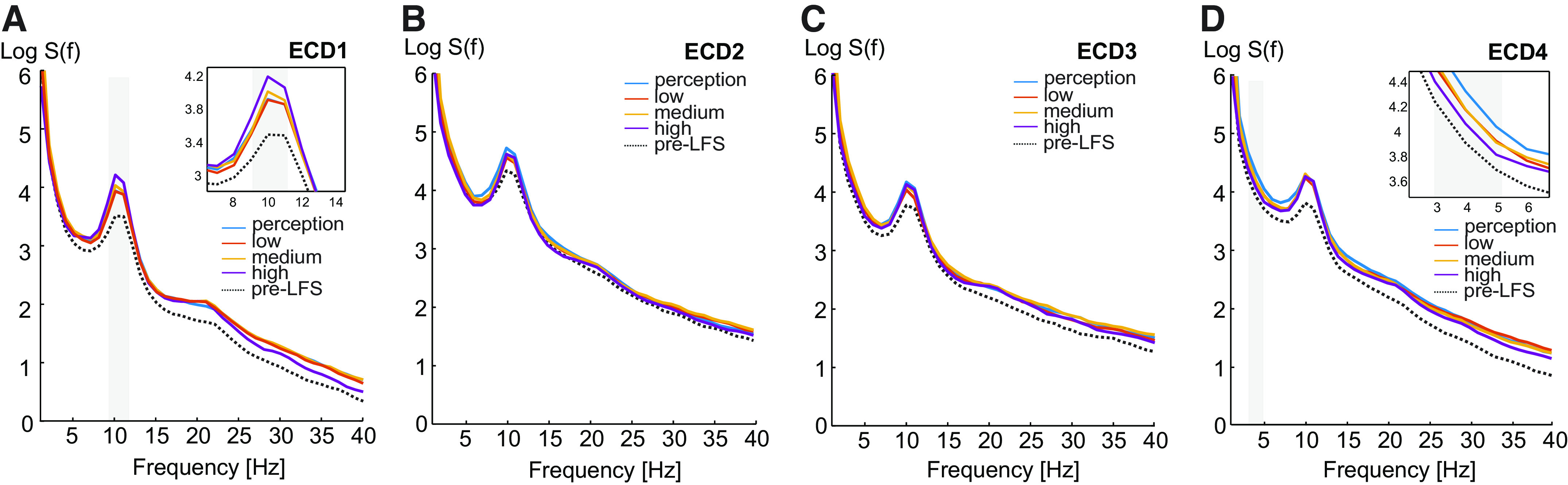
Grand average power spectral densities in resting electroencephalography (EEG) before low-frequency stimulation (LFS) (pre-LFS) and following each LFS intensity block (perception, low, medium, and high) for the four equivalent current dipoles (ECDs) (*A*–*D*). One-way ANOVAs showed a significant effect of LFS intensity on spectral power in ECD1 at 9–11 Hz, and in ECD4 at 3–5 Hz. Log *S*(*f*), logarithmic power spectral density. *n* = 24 (11 females).

Figure 5 shows the power spectral densities in the resting EEG in each of the ECDs before any LFS stimulation (pre-LFS) and after each of four LFS intensity blocks. Resting EEG recorded before LFS is shown only for comparison purposes. Spectral power of ECD1 was significantly modulated by LFS intensity at 9–11 Hz [*F*(3,69) = 3.07, *P* = 0.034, ${\text{η}}_{\text{p}}^{2}$ = 0.12; Fig. 5*A*]. Pairwise comparisons showed that this effect was due to an increase in band power after the strongest LFS intensity, compared with after perception (*P* = 0.038) and low intensities (*P* = 0.001). ECD2 and ECD3 showed no statistically significant changes in spectral power after each intensity of LFS (*P* > 0.05; Fig. 5, *B* and *C*). ECD4 showed a statistically significant decrease in 3–5 Hz power with LFS intensity [*F*(3,69) = 3.24, *P* < 0.027, ${\text{η}}_{\text{p}}^{2}$ = 0.24; Fig. 5*D*]. This effect was due to a significant decrease in power after the strongest LFS intensity compared with perception intensity (*P* = 0.014).

Pearson’s correlations were conducted to analyze the relationship between linear regression slopes in ECD1 and ECD4 at each of the four LFS intensities and normalized change in resting oscillatory band-power after stimulation. Pearson’s correlations showed no statistically significant relationship between the slope of regression in ECD1 and 9–11 Hz normalized band power in ECD1 (*P* > 0.05), or between ECD4 and 3–5 Hz normalized band power in ECD4 (*P* > 0.05), at any of the four LFS intensities.

In summary, results show that LFS delivered at strong intensities (15 times detection threshold) was followed by increased resting 9–11 Hz band power in ECD1 and decreased 3–5 Hz power in ECD4.

## DISCUSSION

The present study investigated the temporal profiles of cortical activity during four intensities of LFS and amplitudes of cortical oscillations following LFS. SEPs related to LFS of the radial nerve were modeled by four ECDs located in contralateral SI/MI, bilateral operculo-insular cortex, and MCC. Source activity in the MCC decreased linearly during LFS, with greater attenuation at increasing LFS intensities. Source activity in ipsilateral operculo-insular cortex also decreased linearly during LFS, albeit only during the two lowest stimulus intensities. In contrast, contralateral SI/MI showed a linear increase of source activity during LFS. Blocks of strong LFS intensities were followed by increased 9–11 Hz α band power in contralateral SI/MI and diminished 3–5 Hz θ band power in MCC.

Diminished source activity in MCC was observed during LFS intensities at greater than perception level. This furthers our previous findings (34), showing that changes in MCC are found at noxious intensities. Involvement of the MCC in somatosensory processing is well established; the MCC is engaged during the anticipation (53) and experience of acute experimental pain (54–57), as well as during nonpainful somatosensory stimuli (58–60). The MCC generates greater activity during noxious stimuli when defensive motor actions are required (61), and connections with the premotor cortex and intralaminar thalamic nuclei have been suggested to mediate nocifensive behaviors (55). Thus, the MCC has been proposed to act as a hub between affective processing, pain, cognitive control, and motor planning (62, 63). Engagement of the MCC during LFS has relevance for neuropathic pain treatments; motor cortex stimulation for neuropathic pain has been shown to increase cerebral blood flow in regions including the cingulate gyrus (64), and transcranial magnetic stimulation over medial scalp regions corresponding to the MCC decreases ratings of noxious electrical stimulation (65). Taken together, decreased MCC responses during LFS at intensities greater than perception may reflect reduced engagement of cingulate nociceptive pathways, which are implicated in neuropathic pain.

Poststimulation amplitude of θ oscillations in the MCC were lower after periods of strong LFS. Theta oscillations have been reported to encode the intensity of acute pain and touch stimuli (66), whereas augmented θ power has been observed in patients with neurogenic pain (67–69), fibromyalgia (70, 71), and primary dysmenorrhea (72). As a result, a shift from dominant α to θ oscillations has been suggested as a contributing factor in the maintenance of chronic pain (73, 74). Greater reduction in power with increased LFS intensities suggests an inhibition within the region of MCC. This reduction in power may be related to the presence of LTD, which is a proposed mechanism of pain suppression during LFS (11, 28). Thus, reduced θ band power after LFS could reflect a reduction of aberrant processes within the MCC that potentially contribute to persistent pain states.

A novel finding in the current study was increasing slopes of activation in SI/MI during LFS, which were enhanced with greater LFS intensities. The primary somatosensory cortex has been implicated in sensory-discriminative processing of noxious and innocuous stimuli, with preferential responses for nonpainful stimulus onsets (59, 75). Pain-related activation has also been demonstrated in the primary motor cortex (57, 76–78). Primary somatosensory cortex activation has been shown to be more resilient to stimulus repetition than other somatosensory processing regions, particularly in area 3b (79–81); although some studies have reported reduced primary somatosensory cortex responses to repeated noxious stimuli (82). Increased amplitude of short-latency SEPs generated in primary somatosensory cortex have been reported alongside increased motor cortex excitability with high intensities of peripheral electrical stimulation, whereas lower stimulation intensities reduce SEP amplitude and cortical excitability (83, 84). Greater cortical excitability in corticomotor pathways after high intensities of peripheral stimulation could mask subsequent parallel inputs via an inhibitory gating mechanism (48, 85). Findings of increased primary somatosensory cortex activation suggest that the analgesic effects of LFS that have been reported in previous studies (28–32) may result from a combination of MCC attenuation and SI/MI facilitation.

Poststimulation amplitude of α oscillations in SI/MI showed increased power after the strongest LFS intensity. Tactile and peripheral nerve stimulation are associated with amplitude attenuation of cortical 10 and 20 Hz oscillations focused primarily over contralateral sensorimotor regions (37, 86–91), followed by an amplitude increase or rebound after stimulus cessation (37, 89, 92–97). Poststimulus increases in μ rhythm, particularly in the 20-Hz component, have been linked to the involvement of the dorsal column pathway (87). Notably, periods of increased oscillatory band power over sensorimotor cortical areas have been found following administration of γ-aminobutyric acid (GABA) agonist benzodiazepines (98, 99), pointing toward a direct role in sensory gating and inhibition (100). Prestimulus α power has been shown to be related to noxious processing, with weaker 10–12 Hz α band power associated with higher amplitude laser-evoked potentials (101). Greater α band power is associated with cortical inhibition (86, 102). Combined, the results suggest that increased source activity in SI/MI during conditioning may inhibit poststimulation processing, due to the possible masking of nociceptive processing by afferent impulses conveyed in ascending spinal pathways.

Ipsilateral operculo-insular source activity decreased selectively during the two lowest LFS intensities, pointing toward a differentiation between noxious and nonpainful stimulation. Operculo-insular cortex has been consistently implicated in pain processing (59, 78, 103–106), with increased activation alongside heightened acute pain perception (107, 108). Similar to conditioning stimuli in the current study, repeated stimuli presented at regular intervals manifest in diminished SEPs as early as the second repetition (109–112). Notably, SEPs during medium and high LFS intensities were reduced during the first minute of stimulation in contrast to the two lowest intensities. Therefore, selective attenuation of operculo-insular source activity during weaker LFS intensities may be due to immediate suppression of evoked potentials during strong stimuli, which show no further decreases during conditioning.

Activity-dependent synaptic plasticity such as LTD have been interpreted as hallmarks of learning and memory (113–115), leading to the suggestion that LTD may be a process for erasing pain memory traces (32, 116). LFS was associated with source activity in the SI/MI, bilateral operculo-insular cortex, and MCC, consistent with previous studies of SEPs elicited by electrical stimulation (117–120) and our previous LFS study (34). Although the contralateral operculo-insular cortex was not affected by intensity, activity in the SI/MI, ipsilateral operculo-insular cortex, and MCC showed greater source activity as LFS intensity increased. This is in line with evidence that graded intensities of nonpainful and noxious stimuli are associated with enhanced amplitude of evoked potentials (110, 118, 119, 121, 122) and greater hemodynamic responses in somatosensory processing regions (57, 78, 108, 123). These findings further previous evidence of reductions in pain-related cortical activation after LFS in regions including the primary and secondary somatosensory cortices, insula, anterior cingulate cortex, and inferior parietal lobule (124).

The present study did not identify significant changes in pain or unpleasantness of electrical test stimuli between LFS intensities. Previous studies have reported a decrease in behavioral pain ratings to noxious stimuli after LFS (28–30), with the strongest attenuation after an intensity corresponding to 15 times detection threshold (29). Such decreases have been interpreted as correlates of LTD of nociception in humans (28). Inconsistencies may be due to variations in methodology; Jung et al. (29) studied the effects of LFS intensity on mean pain ratings from 0 to 100 during eight blocks of 15 test stimuli over 1 h following conditioning stimulation, in comparison with single test stimuli rated from 0 to 10 in the present study. Ratings in the present study may also have been affected by participants’ knowledge that LFS intensity was the same for each test stimulus, as cognitive processes such as expectation are well established as modulators of pain experience (125).

A caveat of the present findings is in the use of source dipole modeling to estimate cortical generators of the observed scalp data. Source dipole modeling is an inverse solution which assumes that the scalp generated field is generated by only one or a few equivalent current dipoles in the brain (126). Studies examining the accuracy of dipole source localization methods have reported mean errors of 6–20 mm (127–130). However, definitive identification of electrical potentials is not possible from EEG alone, and caution should be taken when interpreting the spatial locations of results.

The current findings have potential implications for neuropathic pain treatment. Although preliminary investigations have demonstrated success with LFS (9, 10, 131), evidence is limited, and a recent randomized controlled trial in chronic peripheral nerve injury found no significant reduction in spontaneous pain symptoms following 3 mo of LFS treatment (132). However, these findings may have been affected by stimulation intensity, as patients were able to vary stimulus parameters including intensity as desired, which may have resulted in intensities below that required to activate Aδ fibers (11, 15). A mechanistic arm of the aforementioned trial reported significant effects of LFS on mechanical pain sensitivity and dynamic mechanical allodynia (132). Indeed, LFS has been shown to reverse and inhibit the development of primary hyperalgesia evoked by HFS, even at very low frequencies that would not independently result in LTD (32). This has particular relevance for neuropathic pain, with a large proportion of patients with peripheral neuropathic pain exhibiting mechanical hyperalgesia (133).

### Conclusions

Our study demonstrates that LFS of radial nerve fibers elicits graded effects on somatosensory processing, most notably in the MCC and SI/MI. Although previous literature points toward LTD as the neurophysiological mechanism underlying LFS, our study suggests a potential secondary mechanism involving engagement of the SI/MI. Preliminary findings of a modulation of cortical oscillations after conditioning supports sustained changes after LFS; however, the link between short-term changes in neural activity and long-lasting effects of LFS on persistent pain states has yet to be explored.

## GRANTS

This work was supported by the Medical Research Council under Grant Number MR/P015824/1. 

## DISCLOSURES

No conflicts of interest, financial or otherwise, are declared by the authors.

## AUTHOR CONTRIBUTIONS

D.H. and A.S. conceived and designed research; D.H., A.N.-F., and J.H. performed experiments; D.H. and A.S. analyzed data; D.H. and A.S. interpreted results of experiments; D.H. and A.S. prepared figures; D.H. and A.S. drafted manuscript; D.H., A.N.-F., J.H., N.B.F., C.B., and A.S. edited and revised manuscript; D.H., A.N.-F., J.H., N.B.F., C.B., and A.S. approved final version of manuscript.
